# Advancements in the Management of HPV-Associated Head and Neck Squamous Cell Carcinoma

**DOI:** 10.3390/jcm4050822

**Published:** 2015-04-24

**Authors:** Ross Zeitlin, Harrison P. Nguyen, David Rafferty, Stephen Tyring

**Affiliations:** 1 University of Florida College of Medicine, 1600 SW Archer Rd., Gainesville, FL 32603, USA; 2 Baylor College of Medicine, 1 Baylor Plaza, Houston, TX 77030, USA; E-Mail: Harrison.p.nguyen@gmail.com; 3 Paul L. Foster School of Medicine, 5001 El Paso Dr., El Paso, TX 79905, USA; E-Mail: david.rafferty@ttuhsc.edu; 4 Department of Dermatology, University of Texas Medical School at Houston, 6655 Travis Street, Houston, TX 77030, USA; E-Mail: styring@ccstexas.com

**Keywords:** human papillomavirus, head and neck squamous cell carcinoma, oropharyngeal carcinoma, viral carcinogenesis

## Abstract

Head and neck carcinomas have long been linked to alcohol and tobacco abuse; however, within the last two decades, the human papillomavirus (HPV) has emerged as a third etiology and is specifically associated with head and neck squamous cell carcinomas (HNSCC). In this anatomical region, the oncogenic HPV-16 mediates transformation and immortalization of epithelium, most commonly in the oropharynx. Nevertheless, the recent identification of novel HPV mechanisms thought to be specific to oropharyngeal carcinogenesis has coincided with observations that HPV-associated HNSCC has differing clinical behavior—in terms of natural history, therapeutic response, and prognosis—than HPV-negative head and neck tumors. Taken together with the growing incidence of HPV transmission in younger populations, these discoveries have sparked a rapid expansion in both laboratory and clinical studies on the infection and disease. Herein, we review the clinical characteristics of HPV-associated HNSCC, with particular emphasis on recent advancements in our understanding of the management of this infectious malignancy.

## 1. Introduction

With an annual incidence of nearly 600,000 cases, head and neck carcinomas constitute the sixth most common cancer group worldwide and account for more than 200,000 deaths each year [1]. Over 90% of head and neck carcinomas are squamous cell carcinomas, originating most frequently in the oropharynx, oral cavity, and larynx. The main carcinogenic factors for head and neck squamous cell carcinomas (HNSCC) include tobacco and alcohol consumption, which can have a synergistic effect and are believed to be responsible for up to 75% of cases [2]. However, within the last two decades the human papillomavirus (HPV) has emerged as a novel etiology of HNSCC. 

A 2013 global statistical analysis demonstrated a 36% overall prevalence of HPV in HNSCC, which is nearly double the figure reported one decade ago, while other sources have reported prevalence rates as high as 90% [3,4]. It is estimated that by the year 2020, the incidence of HPV-positive HNSCC in the US will exceed that of cervical cancer [3]. Moreover, HPV positivity is higher in patients younger than 60, which is likely reflective of the decreasing rates of smoking, as well as the increasing transmission of HPV in younger populations [5]. While the carcinogenic mechanisms of alcohol and tobacco result in frequent DNA mutations in HPV-negative HNSCC, HPV-positive tumors have relatively fewer genetic alterations [6]. 

The high-risk oncogenic HPV-16 is the most common type detected in oropharyngeal HNSCC; an analysis of a Canadian pathology database showed 57% of tonsillar cancer biopsies tested positive for HPV-16 [7]. Transformation-specific HPV-16 transcription patterns are correlated with better prognostic and survival outcomes in patients with oropharyngeal carcinoma (OPC) [8,9]. From mechanistic and prognostic perspectives, HPV-driven tumorigenesis in the oropharynx can be categorized as a heterogeneous disease subset distinct from HPV-negative OPC. However, current guidelines describe OPC treatments regardless of HPV status. As knowledge of the pathogenesis of HPV-driven OPC expands, tailored treatments to this disease subgroup are now under development. This article will provide an overview of HPV-driven OPC, with special emphasis on recent advancements in our understanding of the emergence of novel treatments for this infectious malignancy. 

## 2. HPV-Driven Oropharyngeal Carcinoma: A Distinct Disease Subgroup 

### 2.1. Demographics and Risk Factors

Patients with HPV-positive OPC tend to be younger at time of presentation than that of patients with HPV-negative oropharyngeal tumors [10]. In one study examining the incidence of HPV-positive tonsillar carcinoma, the mean age at diagnosis was 59 years, compared to 68 years in patients with no evidence of the virus [11].

Furthermore, oropharyngeal HPV infection is transmitted through bodily fluids, such as during kissing and oral sex. Individuals with six or more lifetime sexual partners are at an increased risk of HPV-related OPC [12]. Other case-controlled studies have linked HPV-positive OPC with sexual debut at a younger age, lack of barrier use during oral sex, and a history of previous sexually transmitted infection [12]. HNSCC tumors in Caucasians have higher rates of HPV-positivity than those in Blacks [13]. Other lifestyle risk factors, such as reduced teeth brushing and high-frequency use of certain types of mouthwashes, result in disturbance of the normal mouth flora, which leads to reduced barrier function and increased vulnerability for infection [14].

### 2.2. Site Predilection

HPV-driven OPC predominantly arises from the reticulated epithelium of the oropharynx, which lines the crypts of the palatine tonsils and base of tongue [15]. The reason for this site predilection is not fully understood, but it has been postulated that the deep and irregularly shaped crypts within the tonsils may allow for prolonged exposure of the pathogen with the lymphoepithelial tissue [16]. From a molecular perspective, the programmed cell death-1 (PD-1)/PD-1 ligand (PD-L1) immunomodulatory cell signaling pathway has been shown to play a role in creating an “immune-privileged” site to allow for viral infection and immune resistance of established tumors in OPC [17]. 

### 2.3. Primary and Nodal Disease

On initial presentation, oropharyngeal tumors may present with dysphagia, referred otalgia, tonsillar asymmetry, or impaired ability to protrude the tongue. However, patients often will have no pharyngeal complaints, since HPV-driven OPC is more likely to present with sudden identification of locally advanced disease in the neck in the presence of early stage occult disease in the oropharynx. It has been postulated that there may be a differential rate of neoplastic growth between the primary oropharyngeal mass and nodal disease, as the metastatic lesion may have more access to nutrients due to its proximity to vasculature [16].

This presentation of abruptly appearing cervical nodal disease may also be explained by the association between HPV-driven OPC and the presence of large cystic nodal metastases [18]. One study found that these metastases were specific to OPC, especially in the setting of a cancer of unknown primary origin, and that they were associated with HPV positivity [19]. To this end, cystic nodal metastases were associated with larger mean lymph node diameters than in patients without cystic nodes [20]. In this study, the authors also reported a significant correlation between high-risk HPV status and larger overall lymph node size; specifically, the mean cervical node diameter in high-risk HPV-positive patients was 27.3 ± 13.1 mm, while HPV-negative patients demonstrated a mean nodal diameter of 18.0 ± 11.5 mm.

## 3. Treatment

Although HPV testing is performed for prognostic purposes, there is not currently sufficient data to support alteration of therapy based on HPV status. Strategies include definitive radiation therapy (RT) for early stage disease or, for more advanced disease, surgical resection and concurrent chemoradiation therapy with agents such as cisplatin. Given the younger associated age of diagnosis and favorable survival outcomes in HPV-positive patients relative to those who are HPV-negative, it is important to develop treatments to reduce late treatment morbidities while maintaining efficacy. The “de-escalation” strategies have been developed to decrease treatment intensity, with the goals of either replacing chemotherapy with more specifically targeted agents, reducing the chemoradiation dose, or replacing chemoradiation with radiation alone [21]. Moreover, another major approach lies in the development of novel therapeutic vaccines against HPV.

### 3.1. Ongoing De-Escalation Trials

The phase 3 EXTREME trial showed that the addition of cetuximab, a monoclonal antibody that inhibits ligand binding to the epidermal growth factor receptor (EGFR), to the first-line agents of platinum/5-fluorouracil significantly improved overall and progression-free survival (PFS) in patients with recurrent or metastatic HNSCC [22]. A retrospective analysis of this trial demonstrated that cetuximab in combination with these first-line agents improved survival independently from p16 or HPV status, while p16 or HPV status is prognostically favorable in the setting of recurrent or metastatic oropharyngeal carcinoma [23]. The use of anti-EGFR monoclonal antibodies has been shown to indirectly induce an antibody-dependent cellular cytotoxicity, conferring an anti-tumor immune response [24]. Given the positive response in HPV-positive HNSCCs and the cytotoxic T-cell-based anti-tumor effects of anti-EGFR therapies, several recent de-escalation studies have been designed to investigate the replacement of cisplatin with cetuximab.

Radiation Therapy Oncology Group (RTOG) 1016 is a multi-center phase III trial involving 706 patients with Stage III or IV p16-positive OPC with the goal of evaluating the replacement of cisplatin with cetuximab [25]. Patients have been randomized to receive weekly cetuximab or intravenous cisplatin with concurrent accelerated intensity-modulated radiation therapy (IMRT) to 70 gray (Gy). The study will evaluate overall survival, PFS, and acute and late toxicities. The main hypothesis is that radiation therapy in concert with cetuximab will lead to less morbidity and better quality of life measures while maintaining overall efficacy. Similar to RTOG 1016, the United Kingdom phase III De-ESCALaTE (Determination of Epidermal growth factor receptor-inhibitor *vs.* Standard Chemotherapy early And Late Toxicity Events) trial involves 304 patients with Stage III or IV p16-positive oropharyngeal carcinoma who are randomized to conventionally fractionated IMRT to 70 Gy with either cisplatin or cetuximab [26]. The study will evaluate for acute and late toxicities, overall survival, recurrence rates, and quality of life measures. The Trans Tasman Radiation Oncology Group (TROG)-12.01 phase 3 trial is similar to RTOG 1016 but involves 200 patients evaluated for early symptom severity [27].

Another set of trials examines the effect of induction chemotherapy followed by decreased chemoradiation dose in good responders. The Eastern Cooperative Oncology Group (ECOG) 1308 trial is a multi-center, phase II study in 90 patients with Stage III or IV HPV-positive OPC (with p16 immunostaining and HPV *in situ* hybridization) [28]. Patients are randomized to receive induction chemotherapy with cisplatin, paclitaxel, and cetuximab, followed by either IMRT to either 54 Gy or 70 Gy concurrently with cetuximab based on response to induction chemotherapy (e.g., complete responders receive low-dose IMRT). The primary endpoint is 2-year PFS, with preliminary results showing one-year PFS rates of 91% and 87% in the reduced- and standard-dose arms, respectively [29]. Similar to ECOG 1308, the Quarterback trial includes a proposed enrollment of 365 patients with Stage III or IV OPC, nasopharyngeal carcinoma, or cancer of unknown primary origin that are p16- and HPV DNA-positive [30]. The patients will undergo induction chemotherapy and will be randomized to receive either IMRT to 56 Gy or 70 Gy with weekly carboplatin and evaluated for locoregional control and 3-year PFS.

The third major group of trials aims to investigate the role of deintensification of chemoradiation after surgical management in Stage III or IV p16-positive OPC patients. In the ADEPT (Post Operative Adjuvant Therapy De-intensification Trial for Human Papillomavirus- related, p16+ Oropharynx Cancer) phase III study, 496 patients with prior trans-oral resection but current metastatic lymph nodes with extracapsular involvement will be randomized to receive either 60 Gy of radiation alone or radiation with concurrent cisplatin and evaluated for 2-year disease-free survival and locoregional control [31]. In the United Kingdom phase II PATHOS (Post-operative Adjuvant Treatment for HPV-positive Tumors) trial, 88 patients are to undergo trans-oral resection with ipsilateral neck dissection [32]. Based on histopathological features of their HPV-positive OPC, they are stratified into low-, intermediate-, or high-risk groups. The low-risk group will be observed, the intermediate-risk group will be randomized to receive either 50 Gy or 60 Gy with IMRT, and the high-risk group will be randomized to receive 60 Gy with or without cisplatin. Patients will assess swallowing function at 12 months. The phase III ECOG 3311 study with 377 patients is designed similarly to the PATHOS trial [33]. Of note, high-risk patients will instead be randomized to receive 66 Gy with IMRT with or without chemotherapy and will evaluate 2-year PFS rates as well as the incidence of grade 3–4 bleeding rates after surgery.

### 3.2. Therapeutic Vaccines

In younger individuals, vaccination plays an increasing role in prevention strategies for overall HPV-mediated disease, including reduction in the oncogenic risk for OPC. It is believed that HPV-related HNSCC is preventable with vaccination, using either the Gardasil or Cervarix vaccines; however, further studies are needed [34,35]. Cervarix is a bivalent prophylactic vaccine for HPV-16 and -18, and preliminary evidence from a population-based trial in Costa Rica showed a 93% reduction of oral HPV-16 and -18 prevalence four years after vaccination [34]. 

Several vaccination strategies are currently under development in phase I trials, including one peptide and one vector vaccine. A pilot study with five patients diagnosed with advanced HPV-positive HNSCC examined a Trojan peptide vaccine consisting of HLA-I and HLA-II T cell epitopes of HPV16 and Melanoma Antigen E (MAGE-A3) tumor suppressor antigen found in epithelial cell malignancies [36,37]. Four of the five patients recognized both constructs and the treatment regimen had acceptable toxicities. Another phase I trial is evaluating the *Listeria monocytogenes*-based HPV vaccine ADX11-001 in patients with Stage II through IV OPC who have undergone transoral robotic surgery [38].

## 4. Other Future Directions 

In addition to the growth in our understanding of therapy for HPV-driven OPC, several ongoing studies are investigating novel methods of diagnosis and prognostic determination. One such study identified aberrant gene methylation of characteristic regions as being a potential non-invasive marker for detection of HPV infection [39]. One of the benefits of epigenetic-based oral HPV screening is rooted in the fact that genes silenced by hypermethylation are still intact, unlike genes that are inactivated by nucleotide sequence variation. While the sensitivity of this approach is reduced, the specificity and positive predictive value were 100%. Perhaps this detection method will join pap smears in the early surveillance of HPV infection. Since oral infection can transfer to genitalia and *vice versa*, the development of a low-cost, non-invasive diagnostic approach for HPV-positive saliva would have a beneficial effect in reducing incidence of both HNSCC and cervical cancer. One issue that remains unaddressed, however, is whether the detection of HPV in an otherwise asymptomatic individual should be treated. Studies must be done to investigate how other risk factors, such as cigarette and alcohol abuse, along with the presence of HPV in the saliva correlate with development of OPC. Certainly, such a study stratifying various risk factors would be useful in developing an algorithm for clinicians to treat asymptomatic HPV-positive patients who meet criteria for intervention.

Additionally, several markers are under investigation as potential prognostic indicators. Preliminary results from a recent retrospective study suggest that cyclin D1 overexpression is a potential prognostic marker of primary OPC [40]. Similarly, tumor hypoxia regulates many cytokines and angiogenic factors, and the presence of these molecules may represent a worse prognosis in HNSCC [41]. Moreover, secretory leukocyte protease inhibitor (SLPI) gene and protein expression is lower in metastatic *versus* non-metastatic HNSCC, and thus, SLPI may be a potential biomarker for identifying individuals at risk of developing metastases [42]. Detection of this expression pattern could lead to these patients would likely benefit more from aggressive therapy. Finally, another study demonstrated an association between the expression of p27 and tumor response to chemotherapy; further studies of this protein as a biomarker for tumor chemosensitivity are warranted [43,44].

A recent study by the Cancer Genome Atlas profiled the genomes of 279 HNSCC samples, and found that HPV-associated tumors contain mutations in the oncogene *PI3KCA*, a downstream protein that is part of the EFGR signaling cascade [45]. The authors also found loss-of-function mutations in the gene *TRAF1* and amplification of the cell cycle regulator gene *E2F1*. These markers may serve as targets for future therapeutic agents against HPV-driven tumorigenesis.

## 5. Conclusions

HPV is currently the most well-known infectious etiology of malignancy, garnering most of its attention from its role in cervical cancer. However, epidemiological trends appear to point towards a growth of incidence in HNSCC that will usurp cervical cancer by the year 2020 as the most common malignancy associated with HPV. Clinically, the characterization of HPV in individual tumors appears to be critical, since HPV-driven tumors are associated with a distinct presentation and better prognosis relative to HPV-negative OPC. While the identification of specific genes associated with both therapeutic sensitivity and prognosis are still in process, methods are already available to characterize the genetic expression of tumors to design a personalized plan of management. Additionally, it will be important to identify potential patient candidates using this personalized management approach who may benefit from the proposed de-escalation treatment regimens in the future. It should be emphasized that possible candidates are likely to include those with the lowest risk of likely recurrence or resistance to this de-intensified treatment. Lastly, the future likely also holds the development or identification of vaccinations that are capable of immunizing against HPV-mediated carcinogenesis of the oropharynx. Overall, our understanding has progressed tremendously over the last two decades and, in the next two decades, will hopefully continue to expand rapidly in a quest to find a consistently effective cure for this infectious malignancy.
